# Reconstruction of Deep Partial-Thickness Burns With Ovine Forestomach Matrix: Results From a Prospective Observational Study

**DOI:** 10.7759/cureus.105987

**Published:** 2026-03-27

**Authors:** Patrick J Kennedy, Michael Young, Kylie Wentworth, Nidhi Aravapalli, Nicole P Bernal, Ariel Rodgers, Laura Pezzopane, Beth McGuire, Jessica Simon, D. Adam Young, John Loftus

**Affiliations:** 1 Department of Surgery and Comprehensive Burn Center, The Ohio State University Wexner Medical Center, Columbus, USA; 2 Department of Medical Affairs, Aroa Biosurgery Limited, San Diego, USA

**Keywords:** burn surgery, deep partial thickness burn, extracellular matrix, ovine forestomach matrix, trauma surgery

## Abstract

Maximizing the critical goals of burn care (efficient healing, pain control, good cosmesis, and patient satisfaction) can reduce hospital burden and enhance patient outcomes. Use of a biologically derived graft in burn management may support these goals by limiting inflammation and infection and improving tissue remodeling; however, rigorous clinical evaluation of these materials is currently limited. This prospective observational study evaluated the safety and performance of a biologically derived graft composed of ovine forestomach matrix (OFM) in promoting the critical goals of burn care in deep partial-thickness burns. Outcome measures included postoperative complications, time to healing, postoperative pain, and a patient and observer scar assessment. The study enrolled 49 burns with a median size of 129 cm^2^ that were treated with a single application of OFM. The median time to healing was 16 days, and the median follow-up period was 164 days. There were no infections observed. The median patient pain score was 3 out of 10 on postoperative day 6, and patient scar satisfaction was 5 out of 5 at long-term follow-up. The majority of treatment areas were scored as having normal pigmentation (n=20; 45.5%), supple pliability (n=37; 84.1%), and flat height (n=33; 75.0%). This prospective observational study suggests that the OFM may support rapid burn healing with minimal pain, good cosmesis, and high patient satisfaction.

## Introduction

Nearly half a million Americans require medical treatment for acute thermal injuries each year [1]. Advances in burn care over the past 40 years have led to a notable improvement in survival, with an expected 97% survival rate in patients admitted to burn centers [2,3]. However, while the acute, lifesaving intervention has greatly improved, maximizing the critical goals of burn care, efficient healing, pain control, good cosmesis, and patient satisfaction remains an ongoing pursuit.

Effective treatment and management of the physical manifestation of burns is often difficult due to the variety of depth, size, and severity. Burn management is further complicated by prolonged healing times [4], altered pharmacodynamics and pharmacokinetics impacting pain management [5], risk of infection [6,7], scarring and contractures [8]. These complications have a significant impact on the long-term aesthetic and functional outcomes of burn patients. Burn injury recovery can also be complicated by the significant post-traumatic mental and emotional distress that patients face [9]. These factors contribute to the significant physical, mental, and financial burden that burn injuries place on patients, their families, and health systems [9-11]. This burden highlights the need for treatments that are both clinically effective and that minimize human and financial resources.

While clinical research has rightly focused on more severe burns, minimal research on deep partial-thickness (DPT) burns has resulted in clinical equipoise, with multiple treatment modalities, limited rigorous investigation in the clinical literature, and disparate protocols across burn centers. While many xenogeneic and synthetic bioscaffolds have been cleared for use in partial-thickness burns, prospective clinical studies in DPT burns are limited. One bioscaffold that has seen recent clinical success is ovine forestomach matrix (OFM), a large decellularized extracellular matrix graft sustainably derived from New Zealand sheep [12]. OFM retains extracellular matrix components, including fibronectin, elastin, fibroblast growth factor, and various other glycosaminoglycans and proteoglycans that support both healing and re-epithelialization [13,14]. Recent studies have reported successful reconstruction using OFM grafts in limb salvage and trauma and acute care surgery [15,16]. For example, a prospective study by Lawlor et al. demonstrated that OFM grafts were effective across 130 complex lower extremity reconstructions, inpatients at risk of amputation, often with only a single product application [15]. In another prospective study, Cormican et al. described the use of OFM to support the development of vascularized tissue coverage for 61 volumetric soft tissue defects, with minimal complications and no graft loss [16]. However, there is limited published data to support the use of OFM grafts in the treatment of burns.

In this prospective observational study, we evaluated the safety and clinical outcomes associated with a single application of OFM grafts in DPT burns. The primary outcome was postoperative complications, while secondary outcomes included time to complete healing, patient-reported pain during early recovery, and long-term scar quality and patient satisfaction.

## Materials and methods

This was a sub-analysis of a larger, observational, multi-center, single arm, Phase IV ongoing Institutional Review Board (IRB)-approved, prospective observational registry (clinical trial registration: NCT05243966) at a large-volume, quaternary-certified burn center, the Ohio State University Wexner Medical Center, Columbus, Ohio, United States. Participants enrolled in the larger registry were those undergoing a range of surgical procedures involving the reconstruction of soft tissues. This sub-analysis is specific to those with DPT burns. The study protocol (Pro00058745) was approved by a central IRB (Advarra, Maryland, USA), and all patients provided written consent for the collection and use of de-identified data. The study was conducted in accordance with the World Medical Association Declaration of Helsinki ethical guidelines. All patients were enrolled between August 2023 and September 2025. 

Inclusion and exclusion criteria are defined in Table 1. All patients included in the analysis were required to have areas of DPT burn injury, as determined by an attending burn surgeon with advanced burn training. Areas of DPT burn were defined clinically by pale wound beds with reduced or absent blanching following initial assessment and/or debridement. Burns were included when the predominant component of the wound bed was clinically consistent with DPT injury as determined by the attending burn surgeon. Because burn injuries commonly demonstrate heterogeneity in depth, limited areas of superficial partial-thickness or full-thickness injury within the wound bed were permitted. However, burns that were predominantly superficial partial thickness or predominantly full thickness were excluded from the analysis, consistent with the study inclusion and exclusion criteria in Table 1. 

**Table 1:** Inclusion and exclusion criteria CDC: Centers for Disease Control and Prevention; OFM: ovine forestomach matrix

Inclusion Criteria	Exclusion Criteria
Willing and able to provide written informed consent and to comply with the requirements of Clinical Investigational Plan	Patients with known sensitivity to ovine (sheep) derived material
Male or female patients aged 18 years or above	Patients with full thickness (‘third degree’) burns
Patients where OFM graft and/or particulate were used as part of their soft tissue reconstruction procedure	Patients with wounds with uncontrolled clinical infection (CDC Contamination Grade=4)
Subjects that are willing and able to comply with all aspects of the treatment and evaluation schedule	Any medical condition or serious intercurrent illness that, in the opinion of the investigator, may make it undesirable for the patient to participate in the study
-	Patient is currently participating or has participated in another clinical study within past 30 days prior to enrollment
-	Pregnant or lactating women
-	Any subject who, at the discretion of the Investigator, is not suitable for inclusion in the study

All procedures were performed in the operating room under general anesthesia. Surgical debridement was performed at the discretion of the attending burn surgeon using standard excision techniques until viable dermis was identified within the wound bed. The method and extent of debridement were determined based on intraoperative findings and institutional standard of care. An OFM graft (2-layer, Myriad Matrix™, Aroa Biosurgery Limited, Auckland, New Zealand) was briefly rehydrated in sterile saline and then applied directly to the wound bed and fixed with staples at each corner. No patients received particulate graft in this cohort. A subgroup of burn wounds additionally received autologous skin cell suspension (ASCS) (ReCell®; Avita Medical, Inc., Valencia, California, United States) in combination with an OFM graft. The use of ASCS was determined by the treating surgeon based on intraoperative assessment and clinical judgment. It was used selectively in cases where additional epithelialization support was considered beneficial, such as burns with areas of deeper dermal injury or high Fitzpatrick’s scores. For these burns, the ASCS was prepared per the manufacturer’s instructions for use and then applied to the debrided wound bed prior to the application of OFM grafts. A non-adherent contact layer was applied over the OFM graft, with water-soluble gel placed on top of the contact layer to maintain a moist wound environment. An absorptive dressing (Sofsorb™; DeRoyal Industries, Inc., Powell, Tennessee, United States) was additionally applied at the discretion of the attending surgeon. A gauze dressing (Kerlix™; Covidien plc, Dublin, Ireland) was placed, prior to compression (ACE™; 3M Company, Maplewood, Minnesota, United States).

The contact layer remained in place for five days postoperatively, with daily outer dressing changes and re-application of the water-soluble gel, as required. Post-operative pain management followed standard institutional burn care protocols using multimodal systemic analgesia as clinically indicated. Specific pharmacologic regimens were determined by the treating clinical team and were not standardized as part of the study protocol. Burns were monitored for complete healing, with healing defined as complete epithelial coverage confirmed by both clinical assessment and automated wound telemetry.

Patient demographics and intraoperative and postoperative assessments were recorded prospectively using an electronic case report form (Tissue Analytics, Net Health Inc., Pittsburgh, Pennsylvania, United States). The primary outcome was postoperative complications. Secondary outcomes included time to complete healing, patient-reported pain, scar satisfaction scores, and observer-assessed scar scales. Patient-reported pain was assessed at follow-up visits using a Numeric Pain Rating Scale from 0 to 10, with 10 representing the worst pain. The custom observer-assessed scar scale evaluated vascularity, pigmentation, pliability, and height, as described by Lawlor et al. [15]. Descriptive statistics (e.g. mean, median) were determined using GraphPad Prism version 9.3.0 (Dotmatics, Boston, Massachusetts, United States). Normally distributed continuous data was expressed as mean and standard deviation (SD), while non-normal data was expressed as median and interquartile range (IQR). Normality of continuous data was determined using the Shapiro-Wilk test.

## Results

The intent-to-treat (ITT) population included 22 burn patients with a total of 49 burns enrolled in this study; all patients sustained burns with areas of DPT injury. Three patients were lost to follow-up prior to the four-week postoperative assessment due to failure to return for scheduled clinic visits. None withdrew consent or experienced adverse events related to treatment. This resulted in a per-protocol (PP) population of 19 patients with a total of 44 burns. Time to heal, pain, and scar outcome analyses were performed on the PP population to ensure adequate follow-up for outcome assessment. In the ITT population, median participant age was 49 (IQR: 28, 64) years and median BMI was 28.8 (IQR: 26.2, 32.1) kg/m^2^ (Table 2). The participants included seven (31.8%) female patients and 15 (68.2%) male patients; four (18.2%) patients were African American/Black, one (4.5%) was Asian, and 17 (77.3%) were White. Eight (36.4%) participants used tobacco, and five (22.7%) had diabetes, two (9.1%) of whom had controlled diabetes and three (13.6%) had uncontrolled diabetes. Two (9.1%) patients had vascular disease. Median percent total body surface area (TBSA) of burns was 9.0% (IQR: 5.0%, 14.0%) (Table 2). Across the cohort, there was a median of two (IQR: 1, 3) burns per patient (Table 2).

**Table 2:** Participant Demographics BMI, body mass index; TBSA, total body surface area; IQR, interquartile range; ITT, intention to treat population; PP, per protocol population

Characteristics		ITT (n=22)	PP (n=19)
Sex, n 9%)	Female	7 (31.8%)	6 (31.6%)
	Male	15 (68.2%)	13 (68.4%)
Age (years), median (IQR)		49 (28, 64)	46 (28, 64)
BMI (kg/m^2^), median (IQR)		28.8 (26.2, 32.1)	29.3 (26.4, 32.6)
Ethnicity, n (%)	Asian	1 (4.5%)	0 (0.0%)
	Black/African American	4 (18.2%)	4 (21.1%)
	White	17 (77.3%)	15 (78.9%)
Tobacco Use, n (%)		8 (36.4%)	5 (26.3%)
Diabetes, n (%)	Controlled	2 (9.1%)	2 (10.5%)
	Uncontrolled	3 (13.6%)	2 (10.5%)
	None, n (%)	17 (77.3%)	15 (78.9%)
Vascular Disease, n (%)	None, n (%)	20 (90.9%)	18 (94.7%)
	Arterial, n (%)	2 (9.1%)	1 (5.3%)
Burns Per Participant, median (IQR)		2 (1, 3)	2 (1, 3)
TBSA (%), median (IQR)		9.0% (5.0%, 14.0%)	9 (4.5, 14)
Burns, n		49	44
Location, n (%)	Lower Extremity	20/49 (40.8%)	18 (40.9%)
	Trunk, n (%)	4 (8.2%)	3 (6.8%)
	Upper Extremity, n (%)	25 (51.0%)	23 (52.3%)
Defect Size (cm^2^), median (IQR)		129 (74, 226)	115 (66, 227)

**Figure 1:**
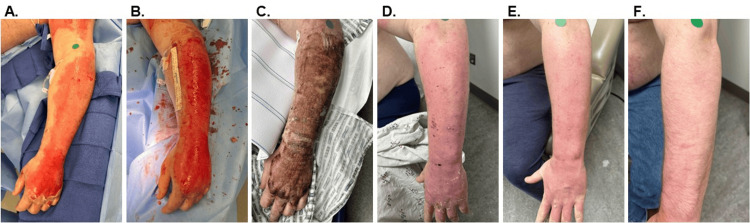
Illustrative case: 30-year-old male patient, left upper extremity DPT burn A. Initial burn presentation. B. After debridement of the defect and prior to OFM graft application. C. Five days after OFM graft application. D. 12 days after OFM graft application. E. 21 days after OFM graft application. F.  Long-term follow-up at 101 days. DPT, deep partial thickness; OFM, ovine forestomach matrix

**Figure 2:**
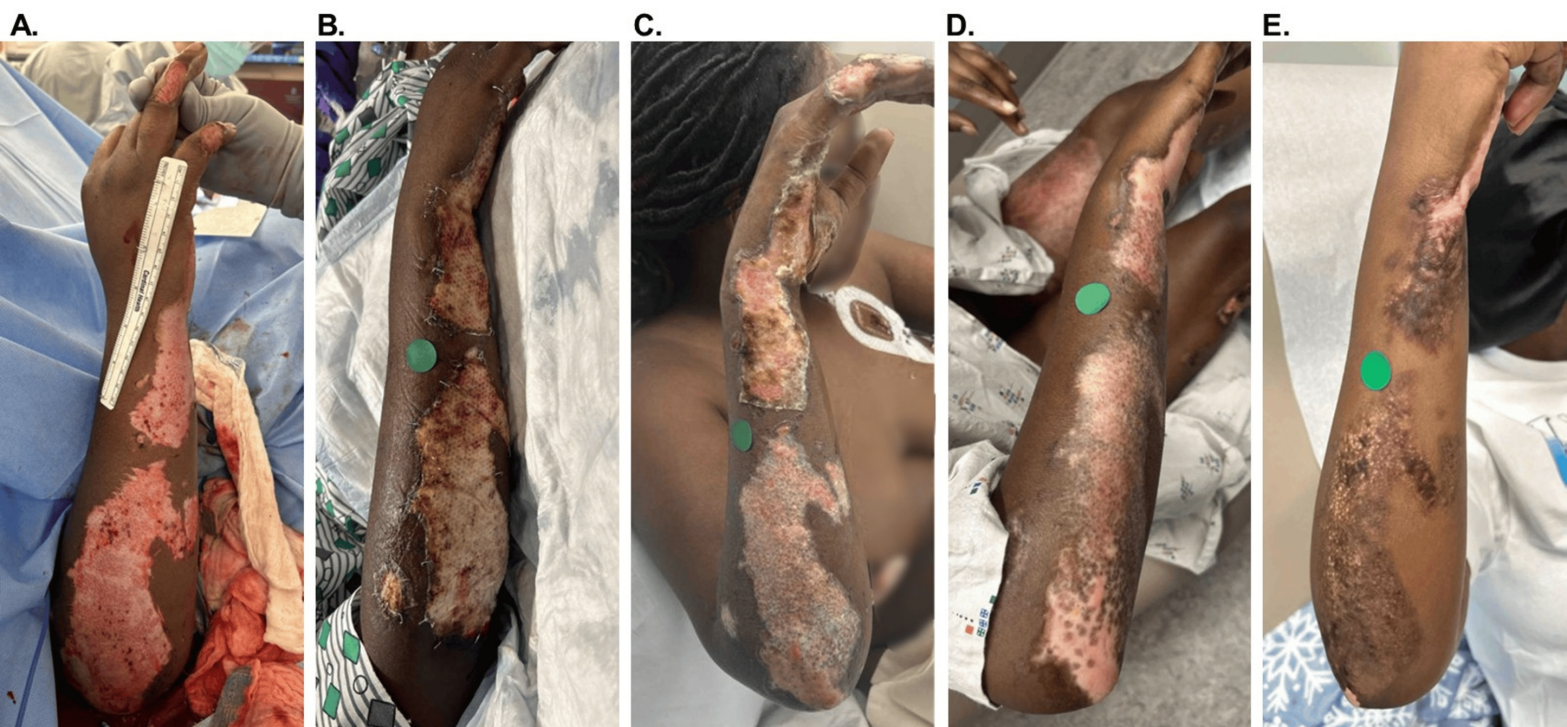
Illustrative case: 24-year-old female patient, right upper extremity DPT burn A. Initial burn presentation. B. Five days after OFM graft application. C. 14 days after OFM graft application. D. 21 days after OFM graft application. E. Long-term follow-up at 170 days. DPT, deep partial thickness; OFM, ovine forestomach matrix

Of the 49 total burns, seven (31.8%) patients had only one burn, seven (31.8%) had two burns, five (22.7%) had three burns, two (9.1%) had four burns, and one (4.5%) had five burns. The median length of hospital stay (LOS) for participants was seven (IQR: 3, 15) days. Illustrative images of patient outcomes are provided in Figures 1, 2. The median burn size was 129 (IQR: 74, 226) cm^2^ (Table 2). Burns were located on the lower extremity (40%), trunk (4%), and upper extremity (51%), with all burns receiving a single application of OFM graft. Six (12.2%) burns additionally received ASCS in combination with the OFM graft.

Included in the PP population were a total of 44 burns with a median follow-up of 167 (IQR: 128, 178) days (Table 3). All burns achieved complete wound closure with the median time to healing of 16 (IQR: 13, 27) days. There were no infections reported across the burns treated with OFM. There were two adverse events reported. One participant experienced a drug eruption, resulting in partial loss (~40%) of the OFM graft that required a split-thickness skin graft (STSG) and was the only defect to require STSG application in the cohort. The defect healed on postoperative day 33. Another participant had a drug eruption secondary to bacitracin and skin staples, though there was no graft loss. The defect healed at postoperative day 13.

**Table 3:** Outcomes IQR, interquartile range; NR, not reported; PP, per protocol population

Outcomes	PP (burns=44)
Number of Applications, median (IQR)	1 (1,1)
Autologous Skin Cell Spray, n (%)	6 (13.6%)
Time to Heal (days), median (IQR)	16 (13, 27)
Maximum Follow-up (days), median (IQR)	167 (128, 178)
Time to Pain Assessment (days), median (IQR)	6 (5, 7)
Patient-Reported Pain Score (out of 10), median (IQR)	3 (2, 5)
Observer Scar Assessment Score [15]	
Scar Vascularity, n (%)	
NR	1 (2.3%)
Normal	11 (22.4%)
Pink	31 (63.3%)
Red	1 (2.0%)
Scar Pigmentation, n (%)	
NR	1 (2.3%)
Normal	20 (45.5%)
Hypopigmentation	6 (13.6%)
Hyperpigmentation	17 (38.6%)
Scar Pliability, n (%)	
NR	1 (2.3%)
Supple	37 (84.1%)
Yielding	4 (9.1%)
Firm	1 (2.3%)
Banding	0 (0.0%)
Contracture	1 (2.3%)
Scar Height, n (%)	
NR	1 (2.3%)
Flat	33 (75.0%)
<2 mm	7 (15.9%)
2-5 mm	3 (6.8%)
>5 mm	0 (0.0%)
Overall Observer Scar Score (out of 12), median (IQR)	2 (1,3)
Time to Scar Assessment (days), median (IQR)	140 (103, 158)
Patient-Reported Scar Satisfaction (out of 5), median (IQR)	5 (4, 5)

Pain score assessment occurred at the protocol-defined one-week postoperative follow-up visit, with the median time of assessment occurring at postoperative day 6 (IQR: 5-7) to reflect patient-reported pain following initial wound stabilization. The median patient-reported pain score was 3 (IQR: 2, 5) out of 10 (Table 3). Participants’ scar assessments occurred at a median time of 140 (IQR: 103, 158) days, with the outcomes presented in Table 3. The median reported patient scar satisfaction was 5 (IQR: 4, 5), on a scale from 0 to 5, with a score of 5 indicating ‘highly satisfied’. The median overall observer scar score was 2 (IQR: 1, 3), using a semi-quantitative scale across four categories, with lower numbers indicative of flat scars with supple pliability and normal vascularity and pigmentation (Table 3). Eleven (22.4%) burns were noted to have normal scar vascularity, whereas 31 (63.3%) had pink, and one (2.3%) had red vascularity. Twenty (45.5%) burns had normal pigmentation, six (13.6%) had hypopigmentation, and 17 (38.6%) had hyperpigmentation. Thirty-seven (84.1%) burn scars were noted to be supple, four (9.1%) were yielding, one (2.3%) was firm, and one (2.3%) was contracted. Thirty-three (75%) burn scars were flat, seven (15.9%) burn scars that were raised less than 2 mm, and three (6.8%) had scars that were 2-5 mm, with no scars measuring greater than 5 mm (Table 3).

## Discussion

As the proportion of patients that survive traumatic burns increases, the clinical focus of burn and reconstructive surgery must shift to improving healing efficiency, pain control, cosmesis, and patient satisfaction, in order to reduce hospital burden and enhance patient outcomes. Our goal in this study was to prospectively assess safety, healing times, early postoperative pain scores, and long-term scar outcomes when utilizing a bioscaffold in the management of DPT burns. In contrast to superficial partial thickness burns that typically can heal on their own in approximately three weeks, DPT burns require deeper debridement into the reticular dermis and risk hypertrophic scarring, contracture, and delayed healing if not properly managed [17]. OFM grafts have recently shown promising outcomes when used in trauma and acute care surgery due to their ease of use, low infection rate, and clinical value [16]. In particular, it has been shown clinically to support both vascularized tissue infill in traumatic defects [18], as well as closure via epithelial migration in more superficial injuries [19]. The ability to serve as both a scaffold for neodermis formation where needed and simultaneously support re-epithelialization could be particularly advantageous in the context of DPT burns.

Our prospective study is the first to demonstrate the safety and clinical outcome of utilizing OFM grafts in DPT burns from a sequentially treated cohort. The primary endpoint of the study was postoperative complications, and across the 49 burns included in this analysis, no infections were reported. One partial graft loss was reported following a drug eruption, which ultimately required a STSG to a small area of the burn. In the PP population, there were a total of 44 defects, all of which healed at a median of 16 days, and no other patients required a STSG. These results indicate that a single application of OFM may have contributed to consistent healing and favorable outcomes, which have important implications for operative efficiency, resource utilization, and patient experience in high-volume burn centers.

Early postoperative pain control is another challenge in DPT burn management, particularly during the first week following surgical debridement and graft application. In this cohort, patient-reported pain scores were low at a median of six days postoperatively, with a median score of 3 out of 10. Early pain assessment was undertaken in this study as this corresponds to the period following initial wound stabilization and early epithelialization, when pain related to inflammation, damaged nerve endings, and dressing changes is often most pronounced. While this data does not prove a causal relationship between OFM and pain reduction, the observed pain scores suggest that treatment with OFM is associated with a favorable early postoperative pain profile in this real-world cohort. Few prospective studies of bioscaffolds in burn care have reported standardized early postoperative pain outcomes, making this an important and patient-centered contribution.

Both observer-reported and patient-reported scar outcomes were favorable at long-term follow-up (140 days, IQR: 103, 158) after OFM application. Observer scar assessments demonstrated predominantly flat scars with supple pliability and acceptable vascularity and pigmentation, while patient-reported scar satisfaction scores were uniformly high. Pigmentation changes are frequently observed during early burn scar remodeling following DPT burns. Hyperpigmentation may gradually normalize over time as scars mature, and therefore, the pigmentation outcomes reported here should be interpreted within the context of the relatively early follow-up period. The alignment between objective scar assessments and patient-reported satisfaction suggests that the observed scar characteristics translate into meaningful patient-perceived benefits. Meanwhile, hypertrophic scars, characterized by reduced pliability and disordered pigmentation, are associated with contractures and functional limitations, including a restricted range of motion and a higher likelihood of subsequent reconstructive procedures [20].

While OFM has shown clinical success in many applications, there is currently limited evidence supporting its use in burn surgery. There is only a single case report describing the use of OFM in the treatment of facial thermal burn injuries [21]. Notably, in this study, the authors reported a decrease in patient-reported pain scores and restoration of similar skin pigmentation postoperatively, aligning with the results of our prospective analysis in DPT burns. Interestingly, there have also been two recent case reports of the use of OFM in the management of autoimmune bullous diseases (AIBDs), namely pemphigus vulgaris and Stevens-Johnson syndrome (SJS)/toxic epidermal necrosis (TEN), which are also commonly seen in burn clinics [19,22]. Both cases reported a reduction of pain and re-epithelialization of the defects within one to two weeks. Preclinical studies have demonstrated that OFM is an inhibitor of matrix metalloproteinases and neutrophil elastase, while also supporting keratinocyte and epithelial cell migration and proliferation [23,24]. OFM has also been shown preclinically to significantly increase angiogenesis and vessel density, particularly compared to other xenografts such as small intestine submucosa [25]. While this study did not establish a comparative effect between standard of care and OFM, these results suggest that the positive clinical outcomes observed may be associated with OFM’s ability to modulate the local inflammatory environment while also supporting re-epithelialization.

While there are now a growing number of different synthetic and tissue-derived bioscaffolds available for use in soft tissue reconstruction, only a handful have been described for the management of burns, and more specifically for DPT burns without autografting, which makes benchmarking with the outcomes of our study challenging. A synthetic bioscaffold, comprised of chemically crosslinked chondroitin sulphate and reconstituted collagen, has been in clinical use for several decades after being first described by Yannas and Burke [26]. Since then, numerous reports have been presented demonstrating the clinical effectiveness of this bioscaffold in full-thickness burns; however, postoperative complications, most notably infection (up to 40%) and graft loss (up to 30%), are a common limitation [27-29]. Notably, there are currently no published clinical studies reporting outcomes specifically in DPT burns as a distinct subgroup with this bioscaffold, with available data derived predominantly from full-thickness or mixed-depth cohorts. A synthetic polyurethane-based bioscaffold has similarly been researched in full-thickness burns to temporize the burn for eventual autograft, but again, there is a dearth of literature describing its use in DPT burns. A systematic review and meta-analysis of this product reported an infection rate of 8.5% [30], while other studies have reported infection as high as 38%, and graft loss has been reported as high as 40% [27,31]. When comparing to other bioscaffolds with data specific to DPT burns, the time to wound closure observed in our study compares favorably. A recent phase 3b study evaluated a bioengineered allogenic cellularized construct in adults with DPT burns, which demonstrated wound closure in 63.5% of patients by 12 weeks, with only approximately half of patients achieving closure by five weeks and 11% requiring subsequent STSG [32]. In contrast, the median time to complete epithelialization in our cohort was 16 (IQR: 13, 27) days, following a single application of OFM. More recently, a prospective study explored a fish skin-derived bioscaffold in 22 patients with DPT burns and reported no infections and a time to re-epithelialization of 17 days; however, the size of the burn treated with fish graft was not reported, and neither were any patient-reported outcomes [33]. While these outcomes are similar to what we have presented in our study, recent publications have suggested that this fish skin graft can be up to five times the cost of OFM [15,16].

Although this was a prospective analysis of burn patients, there were several limitations that must be acknowledged. Burn depth was determined clinically by experienced burn surgeons without the use of adjunctive confirmation, which reflects common practice but introduces potential variability in depth assessment. While all included burns contained areas of DPT injury, some also included regions of mixed depth, which reflects the clinical reality of burn injuries but complicates strict categorization. Additionally, this was a single-arm prospective observational study without a comparator group, limiting conclusions regarding the efficacy of OFM. The sample size was modest and derived from a single center, which may also limit generalizability. Further, a subset of burns in this cohort additionally received ASCS in combination with the OFM graft. While this reflects real-world clinical practice, it may have introduced variation to the epithelialization times, but the small number of cases receiving ASCS limited the ability to perform meaningful subgroup analyses exploring the impact of this addition. Functional outcomes, such as range of motion, were not systematically evaluated in this study. Future studies incorporating functional assessments alongside aesthetic and patient-reported outcomes would provide a more comprehensive evaluation of burn recovery. Because these outcomes include subjective components, the inability to blind evaluators in this study introduced the potential for observer bias. Additionally, while scar outcomes were assessed at a median follow-up of approximately 140 days, burn scar maturation can continue for 12 months or longer. Longer-term follow-up would be valuable to more fully characterize the durability and evolution of scar outcomes. Despite the limitations, these findings show promising initial clinical outcomes and safety for the use of OFM grafts in the treatment of DPT burns.

## Conclusions

While OFM’s efficacy versus standard of care could not be established in the absence of a comparator group, this prospective observational study demonstrates the safety of OFM grafts when used in DPT burns, with healing in ~16 days, minimal pain, good cosmesis and patient satisfaction, and limited need for additional interventions. OFM grafts were placed only once, which may streamline burn management, reduce hospital stays, and enhance patient satisfaction, making it a practical solution for management of DPT burns. Further comparative studies are warranted in DPT and other burn etiologies (e.g. partial thickness) to further validate these initial findings.
